# Physical parameters and biological factors affect the abscopal effect of combining radiotherapy with immunotherapy: an update on preclinical works

**DOI:** 10.3389/fpubh.2024.1517147

**Published:** 2025-01-15

**Authors:** Wangcai Ren, Jialing Wen, Gang Guo, Wenchao Gu, Shenke Zhang, Chang Liu, Kensuke Osada, Takashi Shimokawa, Qiaojuan Wang, Yue Wang, Xuanzhang Tu, Chen Li, Li Sui, Liqiu Ma

**Affiliations:** ^1^Department of Nuclear Physics, China Institute of Atomic Energy, Beijing, China; ^2^National Innovation Center of Radiation Application, Beijing, China; ^3^Department of Artificial Intelligence Medicine, Graduate School of Medicine, Chiba University, Chiba, Japan; ^4^Marshall Laboratory of Biomedical Engineering, School of Biomedical Engineering, Shenzhen University Medical School, Shenzhen University, Shenzhen, China; ^5^Gunma University Heavy Ion Medical Center, Maebashi, Japan; ^6^Department of Accelerator and Medical Physics, Institute for Quantum Medical Science, National Institutes for Quantum Science and Technology (QST), Chiba, Japan; ^7^Department of Radiotherapy and Oncology, The Second Affiliated Hospital of Soochow University, Suzhou, China; ^8^Department of Molecular Imaging and Theranostics, Institute for Quantum Medical Science, National Institutes for Quantum Science and Technology (QST), Chiba, Japan; ^9^China CDC Key Laboratory of Radiological Protection and Nuclear Emergency, National Institute for Radiological Protection, Chinese Center for Disease Control and Prevention, Beijing, China

**Keywords:** abscopal effect, physical parameters, biological factors, radiotherapy, immunogenic cell death

## Abstract

In the process of radiotherapy for cancer patients, there is an extremely low probability phenomenon that the distal tumor/metastasis away from the irradiation field undergoes regression after localized radiation therapy, which is called the abscopal effect. Enhancing the incidence of this phenomenon possesses profound significance for the investigation of metastatic cancer treatment. Currently, the underlying mechanisms of the abscopal effect remain unclear. Radiation-induced immunogenic cell death is considered one of the potential mechanisms for the abscopal effect. From this perspective, we explored how physical parameters and biological factors influence this process. Differences between patients with respect to physical factors and intrinsic biological factors that activate the immune response (acquired factors) may affect the induction of the abscopal effect.

## 1 Introduction

Cancer is one of the most significant diseases affecting human health. According to estimates by the International Agency for Research on Cancer (IARC), one in five individuals worldwide are projected to develop cancer in their lifetime, thus making cancer prevention one of the important public health challenges of the 21st century (1). Radiotherapy, which is recognized as one of the main types of oncological treatment, primarily exerts its tumoricidal effects by disrupting the DNA double-strand of cancer cells, thereby achieving effective control over local tumors (2). The efficacy of radiotherapy for distant metastatic cancers is limited; however, strategies that induce the abscopal effect may increase this efficacy (3, 4).

The abscopal effect, which was first proposed by Mole (5), refers to the phenomenon where in several patients treated with radiotherapy, the volume of distant tumors that are not located within the irradiation field is significantly reduced or tumor regression occurs following local tumor irradiation. Numerous scholars have explored this phenomenon (6–8). A preclinical study in animals indicated that this effect was tumor-specific and not observed in immunodeficient nude mice, thus revealing the involvement of T cells in the mechanism underlying this effect (7). A case report on cutaneous melanoma in 2012 further increased interest in this phenomenon. In this report, after treatment with ipilimumab, the patient’s metastatic chest lesions were subjected to palliative radiotherapy totaling 2,850 cGy; at two months post-radiotherapy, a reduction in the metastatic lesions located outside of the irradiation field was observed. Additionally, changes in the proportions of immune cells and the levels of cell surface antigens in the blood were detected, thereby indicating an enhanced antitumor immune response (8). Thus, the activation of the antitumor immune response may be the key to the induction of the abscopal effect via radiotherapy.

Immunotherapy is known to work synergistically with other therapies to improve treatment efficacy, and RT combined with immunotherapy has been shown to enhance anti-tumor activity (9–11). Active immunotherapy, which enhances anti-tumor immunity by activating the patient’s immune system, has been shown to increase the incidence of the abscopal effect when combined with radiotherapy (12, 13). However, due to the unclear mechanism of the abscopal effect, the key factors that increase the probability of inducing the abscopal effect have yet to be identified.

From this mini review, we discuss the physical and biological factors affecting the abscopal effect and propose potential mechanisms for its induction. Our aim is to provide a scientific basis for optimizing radiotherapy-immunotherapy combination therapy protocols aimed at curing metastatic cancers.

## 2 Physical parameters affect the induction of the abscopal effect

Numerous preclinical studies have revealed the significant impact of various radiation factors, including radiation dose and fractionation, on the efficiency of the abscopal effect. Formenti et al. (14) underscored the importance for researchers to focus on the investigation of the optimal radiation dose and fractionation. A preclinical study performed by the team of the National Institutes for Quantum Science and Technology reported that there is an optimal dose range for the abscopal effect generated with carbon-ion radiotherapy combined with an anti-cytotoxic T-lymphocyte-associated protein 4 (CTLA-4) antibody (15). Yamamoto et al. (16) utilized a mouse model of osteosarcoma to investigate the antitumor effects of photon beam (180 kV, 15 mA) irradiation at different doses and fractions (8 Gy × 3, or 16 Gy × 1) combined with an anti-CTLA-4 antibody on both local and distant tumors. These findings indicated that the combination of high-dose photon beam irradiation with an anti-CTLA-4 antibody significantly enhances the antitumor effect on distant non-irradiated tumors and prolongs the overall survival of mice. Ghaffari-Nazari et al. (17) used a mouse model with a bilateral CT26 colon cancer tumor to assess the impact of different photon beam (6 MV, 3.5 Gy/min) radiation schemes (16 Gy × 1, 10 Gy × 2, and 3 Gy × 10) at biologically equivalent doses on distant non-irradiated tumors. Their study revealed that single high-dose radiotherapy combined with an anti-programmed cell death-ligand 1 (PD-L1) antibody may be the most effective strategy to induce the abscopal effect. However, some preclinical studies have noted that although low-dose photon irradiation is insufficient to directly kill tumors, it can activate and alter the immune environment and modulate the tumor stroma, thereby increasing the efficacy of immunotherapy (18–20). To delve deeper into the influence of dose fractionation on the probability of inducing the abscopal effect, Dewan et al. (21) compared the therapeutic outcomes of different photon beam (6 MV, 600 cGy/min) radiation protocols (20 Gy × 1, 8 Gy × 3, or 6 Gy × 5) in a bilateral tumor-bearing mouse model developed by using murine mammary carcinoma (TSA) cells. Research has revealed that radiotherapy results in comparable control over the primary tumor when combined with an anti-CTLA-4 antibody. Notably, only the fractionated treatments (8 Gy × 3, 6 Gy × 5) successfully induced the abscopal effect. This may be due to the fact that fractionated doses promote the proliferation of effector T cells and reduce regulatory T cell levels, thereby enhancing systemic anti-tumor immunity (22).

In addition to the radiation dose and fractionation, the radiation dose rate is also an important radiation physical parameter. A preclinical study performed by Tinganelli et al. reported that carbon-ion irradiation in ultra-high dose rate (FLASH, >40 Gy/s) reduces lung metastasis in an osteosarcoma mouse model (23). Whether the irradiation dose rate is one of the key factors affecting the induction of the abscopal effect requires further verification by subsequent experiments in the future. Moreover, Liu et al. investigated the abscopal effect induced by different qualities of radiation in a Lewis lung adenocarcinoma tumor-bearing mouse model and reported that, compared with photon radiation, carbon-ion radiation significantly enhanced the abscopal effect (24). These studies suggest that the abscopal effect can be more effectively induced by selecting appropriate radiation physical parameters (such as radiation dose, fractionation, dose rates, and radiation quality, among other factors) for treatment.

## 3 Biological factors affect the induction of the abscopal effect

The induction of the abscopal effect is not only influenced by physical parameters but also significantly affected by biological factors, which can be divided into intrinsic and acquired factors. Intrinsic factors include the genetic background of the host and tumor and the level of tumor immunogenicity, among other factors. These factors form the basis for individual variability in responses to treatment. Acquired factors, on the other hand, involve changes that are induced by therapeutic interventions such as radiotherapy and immunotherapy. For example, radiotherapy can increase the immunogenicity of cancer cells, whereas immunotherapy can activate a systemic antitumor immune response.

As one of the most advanced immunotherapeutic approaches, immune checkpoint inhibitors target checkpoint pathways such as the CTLA-4 and PD-1/PD-L1 pathways, thus promoting the body’s production of an effective antitumor immune response and preventing the immune evasion of tumors. Studies have reported synergistic enhancement of the abscopal effect when immune checkpoint inhibitors are combined with localized radiotherapy (8, 25, 26). The systemic antitumor effect of radiation typically relies on the activation of tumor-specific CD8+ T cells initiated by antigen-presenting cells (APCs). Immunotherapeutic strategies that enhance APC activation are also commonly employed to augment the abscopal effect (27).

The host’s genetic background significantly influences the efficacy of radiotherapy combined with immunotherapy against cancer metastasis. A preclinical study based on carbon-ion radiotherapy combined with dendritic cells (DCs, which are a type of APC) demonstrated that the host’s genetic background has a substantial effect on the effectiveness of combined therapy. Specifically, the therapy effectively suppressed lung metastasis in Th1-dominant mice but was less effective in Th2-dominant mice (28).

The impact of tumor’s genetic background on the abscopal effect has also been investigated. Strigari et al. (29) reported that the abscopal effect induced by photon radiotherapy may depend on the status of the tumor suppressor p53 within the tumor. In an experiment involving the transplantation of wild-type (wt)-p53 or p53-null HCT116 human colon cancer cells into athymic female nude mice, after 20 Gy electron irradiation of the local tumor, non-irradiated wt-p53 distant tumors exhibited significant inhibition of tumor growth, whereas p53-null tumors did not show a noticeable difference.

Furthermore, the immunogenicity of the tumor may also influence the induction of the abscopal effect. Lai et al. (30) categorized tumors into high or low immunogenicity groups based on the number of infiltrating immune cells and the expression of MHC-I molecules. They evaluated different tumor models with varying degrees of immunogenicity (including high and low immunogenicity) via sham irradiation, a single dose of 15 Gy, and three fractions of 5 Gy. The results revealed a positive correlation between tumor immunogenicity and the abscopal effect of radiation therapy. In highly immunogenic tumors (colorectal carcinoma MC38 cells and OVA-expressing EL4 thymic lymphoma E.G7-OVA cells), a single dose of 15 Gy radiation effectively induced the abscopal effect. In poorly immunogenic tumors (Lewis lung carcinoma LL/2 cells and melanoma B16-F10 cells), radiation therapy failed to induce the abscopal effect.

In conclusion, the efficiency of combination therapy in inducing the abscopal effect may be significantly influenced by biological factors such as the genetic background of the host or tumor. Therefore, it is imperative to fully consider the role of these biological factors before initiating clinical studies on the combination of radiotherapy and immunotherapy.

## 4 Potential mechanisms by which physical parameters and biological factors affecting the occurrence of the abscopal effect

The influence of physical and biological factors on the occurrence of the abscopal effect remains an enigmatic subject. Scholars are persistently employing various mouse models to investigate the mechanisms by which radiation therapy induces the abscopal effect, with the goal of elucidating the mechanism of this effect. It is widely accepted that the immune response induced by radiation, which is known as immunogenic cell death (ICD), may be one of the underlying mechanisms for the abscopal effect (31). Radiotherapy can indirectly kill cancer cells by inducing tumor-targeted immune responses. When tumor cells are irradiated and enter the death phase, they transition from a non-immunogenic to an immunogenic state, thereby triggering an antitumor immune response in the body. This process is referred to as ICD (32).

**Figure 1:**
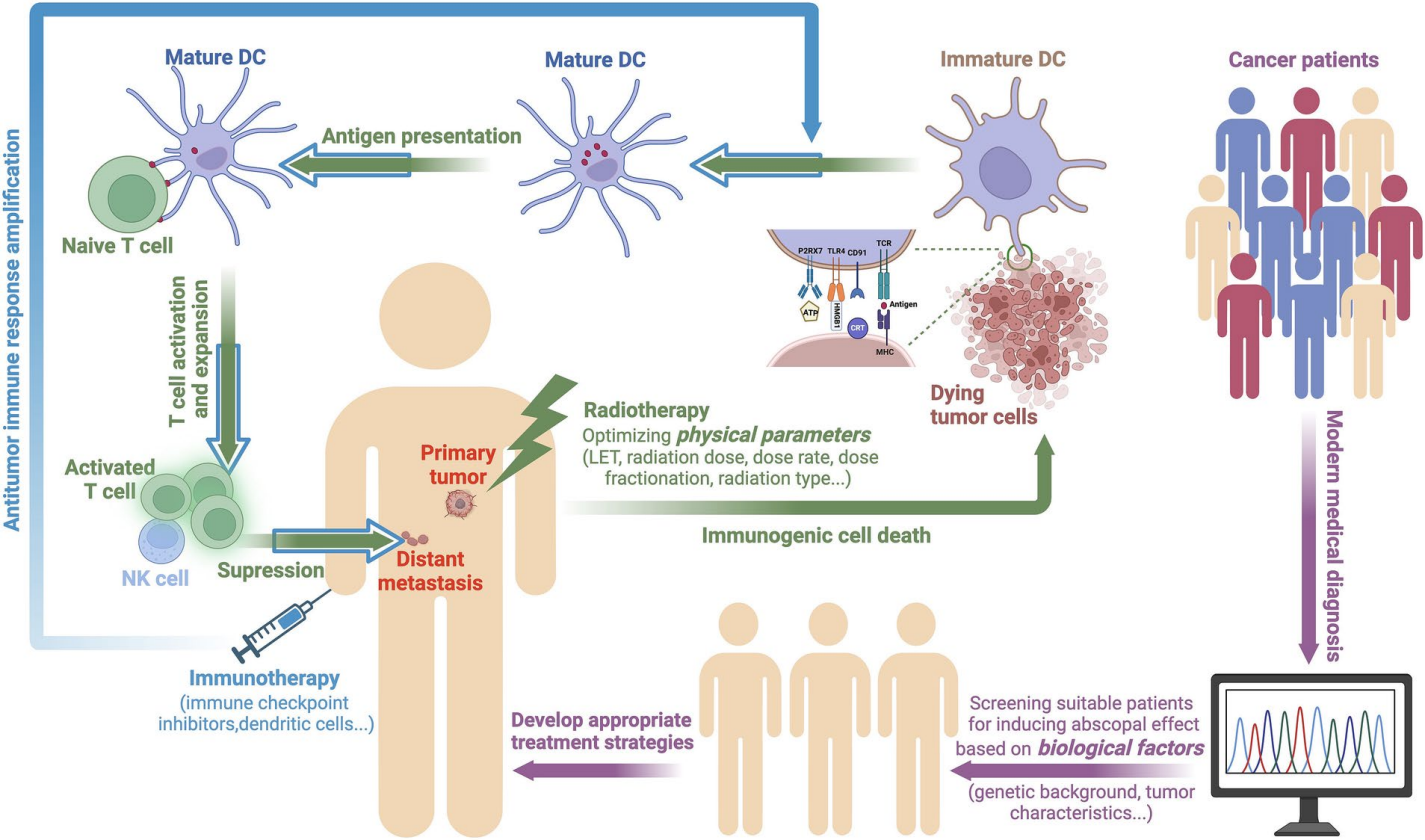
A potential mechanism by which physical and biological factors affect the occurrence of the abscopal effect. For patients possessing abscopal effects that are likely to be induced according to genetic backgrounds and tumor characteristics, optimized radiotherapy may be able to induce ICD in cancer cells, thereby triggering an antitumor immune response. During ICD, cancer cells release DAMPs, such as CRT, HMGB1, and ATP under radiation-induced stress. These DAMPs promote the maturation of DCs, thereby activating T cells to surveil and eliminate tumors. Immunotherapy further amplifies this antitumor immune response, thus extending its therapeutic impact (created with BioRender.com).

During the occurrence of ICD, cancer cells express or release damage-associated molecular patterns (DAMPs), such as calreticulin (CRT), high mobility group protein 1 (HMGB1) and adenosine 5′-triphosphate (ATP), on the cell membrane or release them extracellularly. CRT, which is located on the endoplasmic reticulum, moves to the cell membrane surface as a result of radiation stress and binds to the CD91 receptor on APCs, thereby releasing an “eat me” signal. ATP, which is distributed in the cytoplasmic matrix, is released from inside of the cell to the outside of the cell after radiation, after which it binds to the P2RX7 receptor and functions as a “find me” signal, thereby recruiting dendritic cells, macrophages, and other phagocytic cells to gather at the tumor site. HMGB1 is a non-histone chromatin-binding protein located in the cell nucleus that interacts with DNA and regulates gene transcription. When the cell enters the death phase, HMGB1 is released from the nucleus into the outside, and extracellular HMGB1 activates the corresponding signaling pathways by binding to different pattern recognition receptors (such as TLR2, TLR4, and RAGE), thus promoting the maturation of DCs. Mature DCs “teach” cytotoxic T cells after phagocytosing antigens, which allows them to surveil and eliminate tumors. Additionally, natural killer (NK) cells that are recruited during the ICD process can kill cancer cells by releasing granzymes and perforins (32–34). The *in vivo* antitumor immune activation response that is elicited by radiotherapy plays a vital role throughout the body, thus benefiting patients with metastatic cancer (35, 36). Furthermore, this process is an organic process in which physical factors mediate the response to acquired factors in the body. The increase in the immunogenicity of cancer cells mediated by radiotherapy further promotes the activation of the systemic antitumor immune response, which may induce an abscopal effect, as shown in Figure 1.

A substantial body of preclinical research has indicated that different physical parameters of radiation can have varying impacts on the activation of the biological body’s acquired immune response (Table 1); specifically, different physical parameters can affect the expression levels of DAMPs from tumor cells. Onishi et al. (37) irradiated two types of human cervical cancer cells (HeLa and SiHa) and human esophageal squamous cancer cells (KYSE70) with carbon ion (C-ion) beams at the same biologically equivalent dose but with different LET values (13 keV/μm or 70 keV/μm), and the results revealed that the expression level of HMGB1 in the culture medium at 72 h after irradiation increased with increasing LET. Moreover, the expression levels of DAMPs in various cancer cells exposed to photons (X-ray and *γ*-ray) and proton radiation were observed to change in a dose-dependent manner, whereas there appears to be an optimal dose range in which C-ion radiation induces the maximum expression of DAMPs (38–40).

**Table 1:** The impact of radiation physical parameters on the induction of DAMPs in tumor cells.

Radiation type	Dose rate/LET/energy	Dose	Cancer cell lines	Timepoint	DAMPs	Results	Reference
The impact of LET on the induction of DAMPs in tumor cells							
Carbon ion beam	13 keV/μm	2.8, 3.9, 4.1 Gy	Human cervical cancer cell (HeLa and SiHa) Human esophageal squamous cancer cell (KYSE-70)	72 h	HMGB1	HMGB1 increased with the increase of LET	(37)
	70 keV/μm	1.4, 1.9, 2.3 Gy					
The impact of dose on the induction of DAMPs in tumor cells							
Carbon ion beam	29.1351 keV/μm	2, 4, 10 Gy	Human lung adenocarcinoma (A549) Human glioma (U251MG) Human tongue squamous carcinoma (Tca8113) Human nasopharyngeal carcinoma (CNE-2)	24 h, 48 h, 72 h	CRT	CRT exposure induced by proton beam and X-ray radiation was dose dependent, but C-ion radiation increased the most pronounced CRT exposure at 4 Gy	(38)
Proton beam	1.9779 keV/μm						
X-ray	3.198 Gy/min 2 keV/μm						
γ-ray	5.56 Gy/min	10, 100 Gy	Human breast carcinoma (MDA-MB-231) Human lung carcinoma (NCI-H522) Human prostate carcinoma (LNCaP clone FGC)	72 h	ATP HMGB1	Compared to 10 Gy, 100 Gy radiation significantly increased ATP and HMGB1 expression	(39)
X-ray	600 cGy/min	2, 5, 10, 20 Gy	Murine mammary carcinoma (TSA)	24 h, 72 h	ATP CRT HMGB1	ATP, CRT, and HMGB1 expression levels were altered in a radiation dose-dependent	(40)
The impact of radiation type on the induction of DAMPs in tumor cells							
Carbon ion beam	29.1351 keV/μm	2, 4, 10 Gy	Human lung adenocarcinoma (A549) Human glioma (U251MG) Human tongue squamous carcinoma (Tca8113) Human nasopharyngeal carcinoma (CNE-2)	48 h	CRT	CRT exposure induced by C-ion radiation was most pronounced and time-dependent	(38)
Proton beam	1.9779 keV/μm						
X-ray	3.198 Gy/min 2 keV/μm						
X-ray	0.47–0.50 Gy/min	1, 3, 6, 10, 15, 20 Gy	Murine squamous cell carcinoma (NRS1)	72 h	ATP CRT HMGB1	X-ray and C-ion radiation were equally effective in inducing HMGB1 release, but the CRT level with C-ion radiation was higher than that with X-ray within 10 Gy	(41)
Carbon ion beam	70–80 keV/μm 290 MeV/u						
X-ray	1.1 Gy/min	2.1, 6.7, 8.0, 4.8, 7.1 Gy	Human esophageal squamous cancer (TE2) Human esophageal squamous cancer (KYSE70) Human lung adenocarcinoma (A549) Human large cell carcinoma (NCI-H460) Human colon adenocarcinoma (WiDr)	48 h, 72 h 96 h	HMGB1	X-ray and C-ion radiation at iso-survival doses were equally effective in inducing HMGB1 release	(42)
Carbon ion beam	50 keV/μm 290 MeV/n	0.9, 2.5, 2.7, 1.8, 3.5 Gy					

In addition to dose and LET, radiation type also influences the release of DAMPs. Huang et al. (38) conducted a comparative analysis of CRT following X-ray, proton beam, and C-ion beam irradiation of human tumor cells. These results demonstrated that all forms of radiation led to increased CRT on the cell membrane. Notably, the CRT induced by C-ion beam was most pronounced at the same physical dose (41). Similarly, C-ion beam exposure can induce greater expression of CRT than can photons or protons at the same equivalent biological dose (within a certain range). However, the situation appears to be different for HMGB1. Multiple studies have shown that, compared with X-rays, C-ions are equally effective at inducing HMGB1 release at iso-survival doses (41, 42).

In addition, the biological factors of the body itself can also affect the activation of the acquired immune response under the same radiation physical parameters; specifically, at the same dose and type of radiation, the expression levels of immune-related molecules on the surfaces of tumor cells with different genetic backgrounds still differ. Huang et al. (38) compared CRT exposure on the cell membrane after irradiation of human tumor cells with photons, protons, and carbon ions and reported that the increase in CRT on the cell membrane is tumor dependent. Radiosensitive tumor cells, such as nasopharyngeal carcinoma (CNE-2) and tongue squamous cell carcinoma (Tca-8113) cells, have greater CRT expression on the plasma membrane after radiotherapy than do radioresistant tumor cells, such as glioblastoma (U251) and lung adenocarcinoma (A549) cells; that is, the expression level of immunogenicity-related molecules is affected by the characteristics of the tumor. Such differences may be due to the differences in the immunogenicity of tumor cells under the action of radiation.

In summary, the differences in the efficacy of physical factors and biological factors themselves in activating the acquired immune response may lead to differences in the induction of the abscopal effect in the body. In other words, the key to inducing the abscopal effect may depend on the body’s ability to produce an acquired immune response.

## 5 Discussion and conclusion

To date, research has demonstrated that tumor irradiation can activate an antitumor immune response within the body. However, there is no evidence to suggest that radiotherapy alone can stably induce the abscopal effect (43, 44). The probability of inducing this effect can be increased by combining radiotherapy with immunotherapy, thereby prolonging the survival of patients (45). The primary task that remains in elucidating this mechanism is the determination of the optimal radiation physics parameters, including the type of radiation, dosage, and number of fractions. In addition, the sequence in which radiation and immune drugs are administered can also affect the immune landscape. For example, pretreatment with immune checkpoint inhibitors may help to prime the immune system before radiation exposure, thus potentially leading to a more robust systemic response (46, 47). Conversely, the initial administration of radiotherapy may help to create a favorable environment for subsequent immunotherapy by enhancing antigen presentation and T-cell activation (48). By incorporating contemporary medical diagnostic techniques, the biological characteristics of patients (such as genetic backgrounds and tumor features) are assessed to identify the indications for combined therapy and a suitable patient cohort. Consequently, an appropriate strategy for the integration of radiotherapy and immunotherapy can be formulated to maximize the antitumor effects of both treatment modalities (Figure 1).

Particle therapy is a novel type of efficient radiotherapy that is based on cutting-edge radiation technology. Although particle therapy is gaining traction in cancer treatment, reports on its combination with immunotherapy to induce abscopal effects remain relatively scarce. It is necessary to further strengthen the evaluation of the abscopal effect induced by particle therapy combined with immunotherapy. The exploration of the optimal treatment strategy for the combination of particle radiotherapy and immunotherapy will effectively elucidate the mechanisms underlying the abscopal effect, thus facilitating its transformation from a rare phenomenon to a stable and effective tool for the treatment of metastatic disease.

In conclusion, the combination of radiotherapy with immunotherapy demonstrates great promise for the treatment of cancer. The identification of optimal radiation parameters and the selection of suitable patients for combined therapy are critical steps toward maximizing therapeutic benefits. The potential of the use of particle therapy in combination with immunotherapy to induce the abscopal effect is a novel research field that requires further investigation. Furthermore, a more in-depth understanding of the mechanisms of the abscopal effect will provide a theoretical basis for its clinical application, thus offering a novel and potent approach to combating metastatic cancer.
