# Corrigendum: Physical parameters and biological factors affect the abscopal effect of combining radiotherapy with immunotherapy: an update on preclinical works

**DOI:** 10.3389/fpubh.2025.1561626

**Published:** 2025-01-31

**Authors:** Wangcai Ren, Jialing Wen, Gang Guo, Wenchao Gu, Shenke Zhang, Chang Liu, Kensuke Osada, Takashi Shimokawa, Qiaojuan Wang, Yue Wang, Xuanzhang Tu, Chen Li, Li Sui, Liqiu Ma

**Affiliations:** ^1^Department of Nuclear Physics, China Institute of Atomic Energy, Beijing, China; ^2^National Innovation Center of Radiation Application, Beijing, China; ^3^Department of Artificial Intelligence Medicine, Graduate School of Medicine, Chiba University, Chiba, Japan; ^4^Marshall Laboratory of Biomedical Engineering, School of Biomedical Engineering, Shenzhen University Medical School, Shenzhen University, Shenzhen, China; ^5^Gunma University Heavy Ion Medical Center, Maebashi, Japan; ^6^Department of Accelerator and Medical Physics, Institute for Quantum Medical Science, National Institutes for Quantum Science and Technology (QST), Chiba, Japan; ^7^Department of Radiotherapy and Oncology, The Second Affiliated Hospital of Soochow University, Suzhou, China; ^8^Department of Molecular Imaging and Theranostics, Institute for Quantum Medical Science, National Institutes for Quantum Science and Technology (QST), Chiba, Japan; ^9^China CDC Key Laboratory of Radiological Protection and Nuclear Emergency, National Institute for Radiological Protection, Chinese Center for Disease Control and Prevention, Beijing, China

**Keywords:** abscopal effect, physical parameters, biological factors, radiotherapy, immunogenic cell death

In the published article, there were several errors in affiliations.

Instead of “Shenzhen University Medical School, Shenzhen, China”, affiliation 4 should be “Marshall Laboratory of Biomedical Engineering, School of Biomedical Engineering, Shenzhen University Medical School, Shenzhen University, Shenzhen, China”.

Instead of “Department of Advanced Nuclear Medicine Sciences, Institute for Quantum Medical Science, National Institutes for Quantum Science and Technology (QST), Chiba, Japan”, affiliation 6 should be “Department of Accelerator and Medical Physics, Institute for Quantum Medical Science, National Institutes for Quantum Science and Technology (QST), Chiba, Japan”.

The authors apologize for these errors and state that this does not change the scientific conclusions of the article in any way. The original article has been updated.

